# Quality Assessment of Different Species and Differently Prepared Slices of Zedoray Rhizome by High-Performance Liquid Chromatography and Colorimeter with the Aid of Chemometrics

**DOI:** 10.1155/2020/8866250

**Published:** 2020-09-29

**Authors:** Fei Sun, Xiaolu Yang, Fei Liu, Ying Zhang, Shumei Wang, Hui Cao, Jiang Meng

**Affiliations:** ^1^School of Traditional Chinese Medicine, Guangdong Pharmaceutical University, Guangzhou 510006, China; ^2^Key Laboratory of Digital Quality Evaluation of Chinese Materia Medica, Guangzhou 510006, China; ^3^Engineering Technology Research Center for Chinese Materia Medica Quality of Universities in Guangdong Province, Guangzhou 510006, China; ^4^Guangdong Hexiang Pharmaceutical Co., ltd, Guangzhou 510385, China; ^5^College of Pharmacy, Jinan University, Guangzhou 510632, China

## Abstract

In this study, high-performance liquid chromatography (HPLC) and colorimeter were applied to evaluate the quality of different species and differently prepared slices of Zedoray Rhizome samples with the aid of chemometric tools. Fifty batches of Zedoray Rhizome samples from different species and forty-two batches of Zedoray Rhizome samples from differently prepared slices were collected. The quantitative method was developed using HPLC to simultaneously determine the contents of twelve chemical ingredients in Zedoray Rhizome. The colour parameters *L*, *a*, and *b* were measured by a colorimeter. Then, the collected data were analyzed by the principal component analysis and Pearson correlation analysis. The results showed that the proposed method was capable of accurately determining the contents of the twelve chemical ingredients and the colour parameters for the collected samples. There was a dramatic difference in the contents of the chemical ingredients and in the colour parameters among different species and differently prepared slices of Zedoray Rhizome samples. This study reveals that combining HPLC, colorimeter, and chemometric tools can provide a new approach to comprehensively evaluate the quality of Zedoray Rhizome samples.

## 1. Introduction

Zedoray Rhizome, named as Ezhu in traditional Chinese medicine (TCM), is one of the commonly used Chinese herbs in clinical settings in China. It was firstly recorded in Lei's Treatise on Preparing Drugs and has been listed in the Pharmacopoeia of the People's Republic of China since 1963 [1]. Modern pharmacology studies have shown that Zedoray Rhizome has strong antimicrobial, anti-inflammatory, neuroprotective, anticancer, antiviral, and antithrombotic bioactivities [2–6]. So, Zedoray Rhizome, alone or in combination with other herbs, has been widely prescribed in Chinese clinical practice for the treatment of cardiovascular diseases and cancer.

In Chinese pharmacopeia (2015 edition), Zedoray Rhizome is regulated as the dry rhizome of three species—*Curcuma phaeocaulis* Val. (CP), *Curcuma kwangsiensis* S. G. Lee et C. F. Liang (CK), and *Curcuma wenyujin* Y. H. Chen et C. Ling (CW), and named as “Peng Ezhu,” “Gui Ezhu,” and “Wen Ezhu,” accordingly. These species of Zedoray Rhizome are mainly distributed in China and some other Asian countries. Based on TCM theory, raw herbs need to be processed into small prepared slices for further decoction before clinical usage. As listed in Chinese Pharmacopoeia (2015 edition), the prepared slices of Zedoray Rhizome are classified as two categories—Curcuma Rhizoma (CR) and processed Curcuma Rhizoma with vinegar (PCR), which are rather different in medical efficacy and, therefore, are used for quite different purposes in clinic practice. For example, CR is often used for the treatment of women's blood stasis and amenorrhea, bruises, and food stagnation, while PCR is also used for treating rheumatism as well as shoulder and arm pain in addition to blood stasis and amenorrhea.

Due to the variety in the environment, growth conditions, and processing methods, different species and differently prepared slices of Zedoray Rhizome normally present with distinguished features in the appearance [1] and even chemical ingredients [7, 8]. However, according to the Chinese pharmacopeia (2015 edition), the content of volatile oil has been officially set as the quality assessment criterion for all of the raw and processed herbs of Zedoray Rhizome, and they even share the same quality criterion in spite of the significant interspecies discrepancy and the differences between preparing methods. It is well known that typically, a Chinese herb has multiple chemical ingredients, so only quantifying the amount of volatile oil is not good enough to differentiate the quality of different species and differently prepared slices of Zedoray Rhizome. Therefore, it is urgent to develop a novel approach and establish a more reasonable criterion to evaluate the quality of Zedoray Rhizome samples.

So far, a variety of methods, such as polyacrylamide gel electrophoresis (PAGE), high-performance liquid chromatography (HPLC), gas chromatography (GC), high-performance liquid chromatography-mass spectrometry (HPLC-MS), and gas chromatography-mass spectrometry (GC-MS), have been developed for quality assessment of Zedoray Rhizome. Tang et al. compared the characteristic bands of esterase isozymes among different species of Zedoray Rhizome by PAGE [9]. Yang et al. applied GC-MS to develop the fingerprint of three species of Zedoray Rhizome [10]. Also, Ni et al. constructed the fingerprint of CP, CK, and CW using GC-MS and HPLC, respectively. Besides, three discriminant methods—linear discriminant analysis (LDA), backpropagation-artificial neural networks (BP-ANNs), and least squares-support vector machine (LS-SVM)—were employed and compared with each other to describe the quality for different species of Zedoray Rhizome [11]. Nevertheless, the previous studies mostly focused on the quality assessment of Zedoray Rhizome by developing the chromatographic fingerprint or multi-ingredient quantitative methods, but little attention was paid on the differentiation of the Zedoray Rhizome samples from the perspective of their appearance. Colour is one of the essential appearance features of Chinese herbs. Generally, the colour is evaluated by trained experts. However, the visual colour assessment by naked eyes is easily affected by the environment, illumination, and individual visual difference, which may result in varied conclusions from different evaluators even for the same sample [12]. Recently, the colorimeter is gaining increasing popularity in various fields, such as food quality control and drug identification, due to its simple, fast, and nondestructive nature as a tool for colour identification [13–15]. To date, this technology has also been widely used for quality assessment for Chinese herbs [12, 16, 17]. Nevertheless, solitary colour analysis using a colorimeter without considering the correlation between colour parameters and chemical ingredients of the herbs is the major defect of the traditional colorimeter tests, and cannot fully and objectively reflect the quality of the samples.

Therefore, in this study, a combined approach was developed by integrating HPLC, colorimeter, and chemometric tools for a comprehensive quality assessment of Zedoray Rhizome samples. Totally, fifty batches of Zedoray Rhizome samples from different species and forty-two batches from differently prepared methods were collected. The contents of twelve chemical ingredients were determined by HPLC, and the colour parameters were measured by a colorimeter. Then, the principal component analysis (PCA) [18] was applied to analyze these samples. Finally, the correlation between the colour parameters and the chemical ingredients was analyzed by Pearson correlation analysis.

## 2. Materials and Methods

### 2.1. Materials and Reagents

The fifty batches of different species of Zedoray Rhizome samples were collected from various regions of China, Burma, and Vietnam. All collected samples were authenticated by Professor Jizhu Liu (the School of Traditional Chinese Medicine, Guangdong Pharmaceutical University). Twenty-one batches of CR were purchased from manufacturers who focus on the production of decoction pieces of Chinese herbs. The collected CR samples were authenticated as *Curcuma kwangsiensis* S. G. Lee et C. F. Liang (CK). Then, part of the CR samples from each batch was used to prepare the PCR samples according to the Chinese Pharmacopoeia (2015 edition) [19]. Eventually, a total of twenty-one batches of PCR were well prepared for further analysis. Voucher specimens were deposited at the Herbarium Centre of Guangdong Pharmaceutical University. The details of the collected samples were summarized in Tables 1S and 2S in Supplementary Materials.

**Figure 1:**
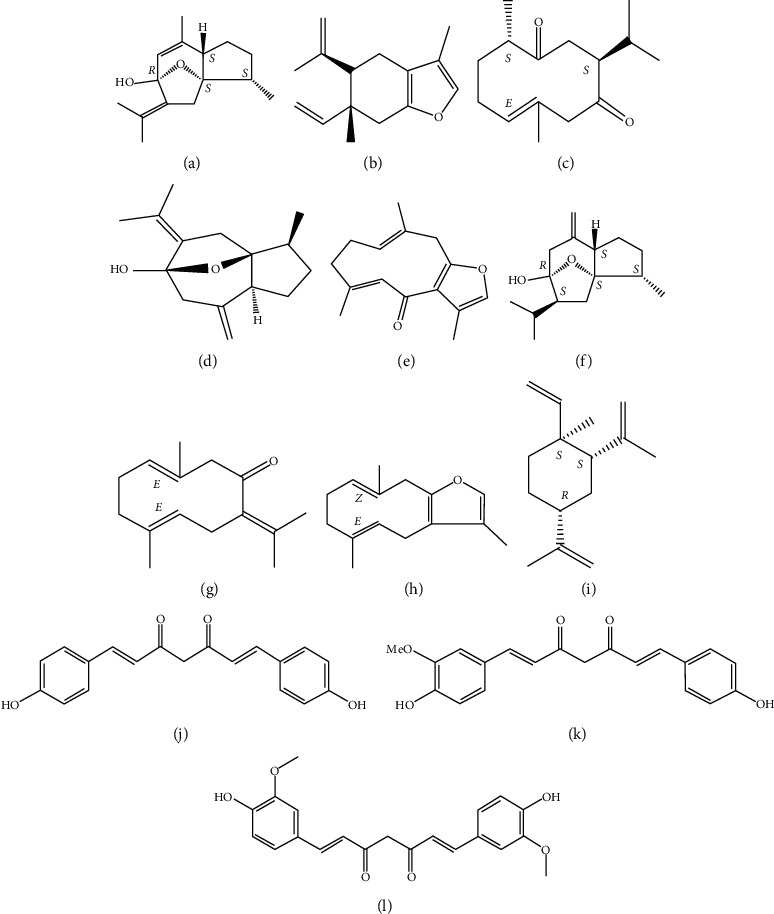
The structure of the standard substances. (a) Curcumenol. (b) Curzerene. (c) Curdione. (d) Isourecumenol. (e) Furanodienon. (f) Curcumol. (g) Germacrone. (h) Furanodiene. (i) *β*-elemene. (j) Bisdemethoxycurcumin. (k) Demethoxycurcumin. (l) Curcumin.

The standard substances were purchased from the following resources—curcumenol and curzerene (Chengdu Herb Purify Co. Ltd., China); isourecumenol, furanodienon, curcumol, furanodiene, *β*-elemene, and curcumin (Chengdu Chroma-Biotechnology Co. Ltd., China); curdione, germacrone, bisdemethoxycurcumin, and demethoxycurcumin (Nanjing Plant Origin Biological Technology Co. Ltd., China). The purity of all the standard substances was above 98%, and their chemical structures are shown in Figure 1. Acetonitrile and methanol (HPLC grade) were obtained from Merck (Darmstadt, Germany), phosphoric acid (HPLC grade) was from Aladdin Biochemical Technology Co. Ltd. (Shanghai, China), and the ultrapure water from A.S. Watson Group Ltd. (Hong Kong, China). Other chemicals used in this study were of analytical grade and were provided by Guangzhou East Giant Experimental Instrument (Guangzhou, China).

### 2.2. Preparation of Standard Solution

The standard substances of curcumenol, curzerene, curdione, isourecumenol, furanodienon, curcumol, germacrone, furanodiene, *β*-elemene, bisdemethoxycurcumin, demethoxycurcumin, and curcumin were accurately weighed and dissolved with methanol to obtain the stock solutions at the concentrations of 3.74 mg·mL^−1^, 7.50 mg·mL^−1^, 7.22 mg·mL^−1^, 0.32 mg·mL^−1^, 6.92 mg·mL^−1^, 5.50 mg·mL^−1^, 2.05 mg·mL^−1^, 10.87 mg·mL^−1^, 3.45 mg·mL^−1^, 0.37 mg·mL^−1^, 0.24 mg·mL^−1^, and 0.26 mg·mL^−1^, respectively. The working standard solutions, after prepared by mixing and diluting the stock solutions with methanol, were filtered through a 0.22 *μ*m PTEE filter. The stock solutions and working solutions were stored at 4°C for further use.

### 2.3. Preparation of Sample Solution

Each batch of the samples was ground and passed through a 180 mesh sieve. The homogenized sample powder (1.0 g) was accurately weighed and extracted with 8 mL of methanol for 45 min by sonication at room temperature. Additional methanol was then refilled to make up the loss. The extracting solution was filtered through a 0.22 *μ*m PTEE filter.

### 2.4. Apparatus and Chromatographic Conditions

The HPLC analysis was carried out with a Shimadzu LC-20AT HPLC system equipped with a diode array detector. The separation was performed on an Ultimate TM XB-C_18_ analytical column (250 mm × 4.6 mm, 5 *μ*m) at 25°C. The mobile phase consisted of a mixture of acetonitrile (A) and 0.2% v/v phosphoric acid in water (B). A gradient program was set as follows: 30% A at 0∼5 min, 30%–60% A at 5–35 min, 60%–68% A at 35–39 min, 68% A at 39–43 min, 68%–80% A at 43–49 min, 80%–95% A at 49–69 min, and 95% A at 69–76 min. The flow rate was 0.8 mL/min, and the detection wavelength was 210 nm and 415 nm. 10 *μ*L of the working solution or the sample solution was injected for HPLC analysis.

### 2.5. Methodology Validation

The linearity of the HPLC method for each analyte was evaluated by calibration curves. Each analyte at a series of different concentrations was analyzed in triplicates. The limit of detection (LOD) and limit of quantification (LOQ) for each of the analytes were determined as signal-to-noise ratio (S/N) of 3 and 10, respectively. The precision of the HPLC method was determined by intraday and interday measurements. The working standard solution was analyzed in six replicates on the same day to obtain the intraday precision while the interday precision was obtained by analyzing the working standard solution daily (six replicates) for three successive days. Meanwhile, the stability was assessed by analyzing the same sample solution (CR10) at 0, 3, 6, 9, 12, and 24 h, respectively. Besides, recovery tests (CR10) were performed according to Chinese pharmacopeia to investigate the accuracy of the developed HPLC method. Mixed standard solutions at the uniform concentration level (100%) were added into 0.5 g of the known real samples, and each solution was done three copies in parallel according to the proposed HPLC method. The results were expressed as relative standard deviation (RSD, %) of the measurements.

### 2.6. Colour Measurement

The colorimeter instrument used in this study consisted of a measuring head (CR-410, Japan), granular attachment (CR-A50), white calibration plate (CR-A44), glass light projection tube (CR-A33e), and colour management software (Spectra Magic NX CM-S100W). Each batch of the samples was milled through a 0.33 mm aperture before measurement to ensure the uniformity of powder. A standard light source was employed to illuminate the samples, and a white calibration plate was used for emendation under artificial daylight conditions (6500 K). Then, the homogeneous samples were put into the granular attachment, and the photoelectric detector was used to monitor the reflected light, which was generated upon selective absorption, reflection, or scattering of the samples. Finally, colour parameters including *L*, *a*, and *b* were calculated by comparing the reflected light with the standard. Colour space of *L*, *a*, and *b* is a colour model set by the International Commission on Illumination (CIE). Parameter *L* represents brightness ranging from the brightest (Δ*L*+) to the darkest (ΔL−). The parameter *a* indicates red (Δ*a*+) and green (Δ*a*−), and the parameter *b* stands for yellow (Δ*b*+) and blue (Δ*b*−). The colour measurement was validated by the precision and stability. The precision of the colorimeter was determined by repeat measurements of the same sample, and the stability was assessed for five consecutive days.

### 2.7. Statistical Analysis

The difference in chemical ingredients and colour parameters between different species and differently prepared slices of Zedoray Rhizome samples was analyzed by the one-way analysis of variance (ANOVA) using SPSS 22.0 software (SPSS Inc., Chicago, USA). Then, the principal component analysis (PCA) was performed to analyze the chemical ingredients and colour data in combination using MATLAB R2009 software (Math Work Inc., South Natick, MA). Finally, the correlation between the chemical ingredients and the colour parameters was analyzed by Pearson correlation analysis with SPSS 22.0 software. *p* < 0.05 was considered statistically significant in this study.

## 3. Results and Discussion

### 3.1. Optimization of Extraction Conditions

To ensure the complete extraction of analytes for HPLC analysis, different extraction methods (sonication or reflux), extraction solvents (various concentrations of aqueous methanol and ethanol), and extraction time (30, 45, or 50 min) were optimized. The results are shown in Table 1. The high yields of analytes were obtained after 45 min of sonication in methanol.

### 3.2. Optimization of Chromatographic Condition

**Figure 2:**
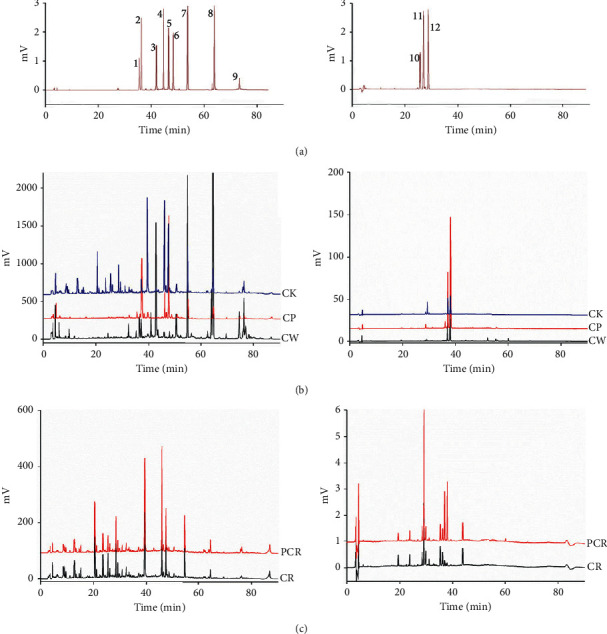
Representative chromatograms of standard substances (a) and samples (b, c) at 210 nm and 415 nm. Peaks 1∼12 refer to curcumenol, curzerene, curdione, isourecumenol, furanodienon, curcumol, germacrone, furanodiene, *β*-elemene, bisdemethoxycurcumin, demethoxycurcumin, and curcumin, respectively.

In this study, different mobile phase combinations were tested. The mobile phase consisting of 0.2% v/v phosphoric acid in water and acetonitrile were successfully used to separate the twelve target analytes. The resolution of the target peaks was over 1.5, while the peak widths were relatively low, and the analysis time was moderated. As a result, a mixture of 0.2% v/v phosphoric acid in water and acetonitrile was selected as the mobile phase. The flow rate was 0.8 mL·min^−1^, and the column temperature was 25°C. The wavelength was set as 210 nm to detect curcumenol, curzerene, curdione, isourecumenol, furanodienon, curcumol, germacrone, furanodiene, and *β*-elemene, and 415 nm to detect bisdemethoxycurcumin, demethoxycurcumin, and curcumin. Under the optimized chromatographic conditions, good baseline, high resolution for target peaks, and reasonable analytical time were warranted (Figure 2).

### 3.3. Methodology Validation

The results of the methodology validation for HPLC analysis are shown in Table 2. The calibration curves of each analyte displayed good linearity over the range of different concentrations. LOD and LOQ were within the range of 0.00668∼0.326 *μ*g·mL^−1^ and 0.0223∼1.085 *μ*g·mL^−1^, respectively. The RSD values of the precision test were 0.19∼1.85% for intraday assays and 0.52∼1.64% for interday assays. The RSD values of stability tests were 0.37∼2.11%. The recoveries of the HPLC method were above 98%, and the RSD values were less than 3.0%. The results demonstrated that the developed HPLC method was capable of accurately determining the contents of the twelve chemical ingredients in different Zedoray Rhizome samples.

### 3.4. Sample Analysis

**Figure 3:**
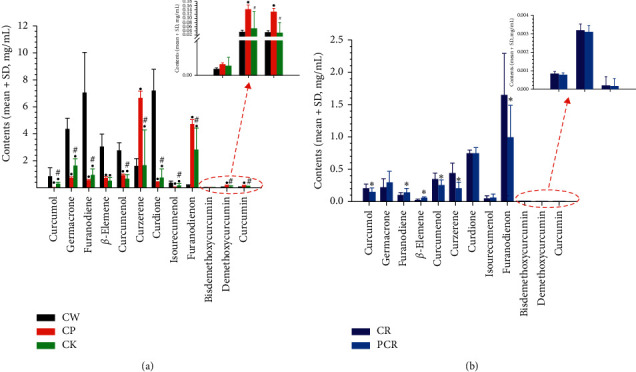
Contents of twelve chemical ingredients in different species (a) and different prepared slices (b) of Zedoray Rhizome samples. ^•^Compared with CW, *p* < 0.05; ^#^compared with CP, *p* < 0.05; ^*∗*^compared with CR, *p* < 0.05.

The developed HPLC method was applied to simultaneously determine the contents of the twelve chemical ingredients in different Zedoray Rhizome samples. The results are shown in Figure 3. There was a significant difference in the contents of the twelve chemical ingredients between different species of Zedoray Rhizome samples (Figure 3(a)). The contents of curcumol, germacrone, furanodiene, *β*-elemene, and curcumenol in CW were much higher than those in CK and CP. Since the previous study has proven that these ingredients have strong antitumor activity, this result may indicate the superiority of CW in this regard when compared with CK and CP. On the other hand, the content of curcumin in CP was significant higher than that in CW and CK, while the levels of curzerene, curdione, isourecumenol, and furanodienon in CP and CK were equivalent and both superior to CW.  Figure 3(b) shows that the contents of curzerene, furanodienon, curcumol, and bisdemethoxycurcumin in CR were higher than those in PCR. As curzerene, furanodienon, curcumol, and bisdemethoxycurcumin contain multiple double bonds, phenolic hydroxyl groups, and carbonyl groups, the levels of these ingredients were decreased due to instability during the processing procedure. By contrast, compared with CR, the contents of germacrone, furanodiene, and *β*-elemene in PCR were increased, indicating the bioactive ingredients of germacrone and *β*-elemene were retained in PCR after CR was processed with vinegar. This provided a theoretical basis for the changes in efficacy before and after CR was processed.

### 3.5. Colour Measurement

**Figure 4:**
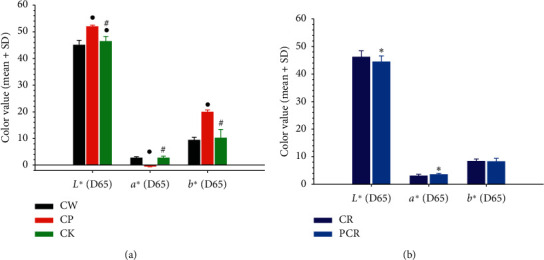
Colour measurement of different species (a) and differently prepared slices (b) of Zedoray Rhizome. ^•^Compared with CW, *p* < 0.05; ^#^compared with CP, *p* < 0.05; ^*∗*^compared with CR, *p* < 0.05.

Before sample analysis, the colorimeter precision and stability were tested. The RSD values of precision and stability were less than 3.0%, indicating the colorimeter was capable of accurately measuring the colour parameters for each sample. The measurements of colour parameter *L*, *a*, and *b* for different Zedoray Rhizome samples are shown in Figure 4. It can be observed that the values of chromatic aberration demonstrated a significant difference between different Zedoray Rhizome samples.

### 3.6. Principal Component Analysis

The PCA model was built on the fused data that combined chemical ingredients and colours for different species of Zedoray Rhizome samples. Before modeling, the fused data were pretreated with the autoscaling method. Generally, the appropriate number of principal components (PCs) should be selected to build the PCA model. Previously, several methods were reported in the literature. In this case, the number of PCs used to build the model was determined with the “eigenvalue-greater-than-one” rule. As a result, the first three PCs that explained 88.5% of the total variance were selected to build the PCA model, as shown in Table 3. The first three PCs accounted for 50.3%, 25.9%, and 12.3% of the total variance, respectively.

**Figure 5:**
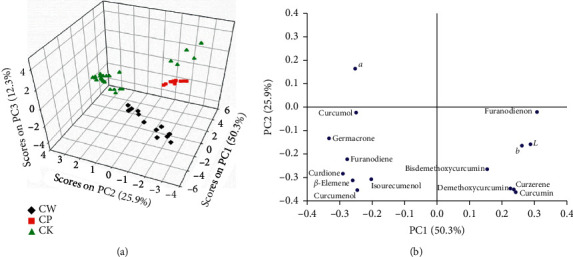
PCA model constructed on the data that combined the chemical ingredients and colour parameters for different species of Zedoray Rhizome samples. (a) Score plot. (b) Loading plot.

Then, the Zedoray Rhizome samples were projected to the PC space to construct the score plot. As is shown in Figure 5(a), the different species were distributed with a gathering trend but located in different position in the score plot. Basically, the CW samples were located in the bottom of the PC space, while the CK samples were in the upper area and the CP species in the middle of the PC samples. This result indicated that the content of chemical ingredients and colour parameters varied between different species of Zedoray Rhizome samples. Furthermore, the distance between the samples in the score plot also reflected the degree of variability in these samples in terms of their original variable space [20]. It could be observed that the variability of CW and CK samples was higher as compared with the CP samples, which was also consistent with the findings in Figure 2(a). The loading plot of the PCA model, shown in Figure 5(b), revealed that curcumol, germacrone, furanodiene, *β*-elemene, curcumenol, curdione, furanodienon, demethoxycurcumin, curcumin, *L*, *a*, and *b* made a major contribution to the first PC. These chemical ingredients and colour parameters presented with similar absolute values of loadings but in the same or opposite directions, indicating that they were positively correlated or inversely related (anticorrelated) with each other. Specifically, parameters *L* and *b* were positively correlated with furanodienon, demethoxycurcumin, and curcumin but inversely related to curcumol, germacrone, furanodiene, *β*-elemene, curcumenol, and curdione. In contrast, the parameter *a* had a positive correlation with curcumol, germacrone, furanodiene, *β*-elemene, curcumenol, and curdione but negatively correlated with furanodienon, demethoxycurcumin, and curcumin.

Also, a PCA model was built on the fused data for differently prepared slices of Zedoray Rhizome samples following data pretreatment with the autoscaling method. Three PCs that accounted for 64.8% of the total variance (34.3%, 18.7%, and 11.8%, respectively) were selected to build the PCA model, as shown in Table 4.

**Figure 6:**
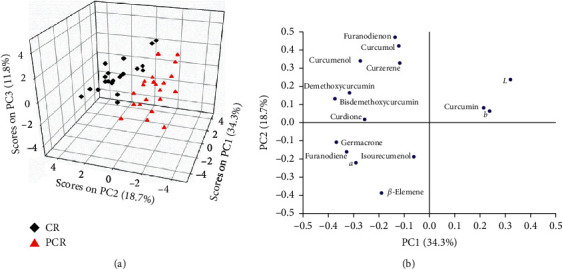
PCA model constructed on the data that combined the chemical ingredients and colours for differently prepared slices of Zedoray Rhizome samples. (a) Score plot. (b) Loading plot.

The score plot of the PCA model is shown in Figure 6(a). Although the CR and PCR samples demonstrated a scattered distribution in the PC space, they could be clearly distinguished from each other in terms of their position, with the CR samples mainly located in the upper, and the PCR in the bottom area of the PC space. The loading plot shown in Figure 6(b) revealed that germacrone, furanodiene, curcumenol, demethoxycurcumin, *L*, *a*, and *b* contributed most to the first PC, indicating that the first PC mainly described the differences in the levels of germacrone, furanodiene, curcumenol, and demethoxycurcumin, as well as in the colour parameters for differently prepared slices of Zedoray Rhizome samples. According to Figure 6(b), parameters *L* and *b* were inversely related to germacrone, furanodiene, curcumenol, and demethoxycurcumin, while the parameter *a* was positively correlated with germacrone, furanodiene, curcumenol, and demethoxycurcumin. The second PC mainly described the differences in curcumol, *β*-elemene, and furanodienon.

### 3.7. Pearson Correlation Analysis

To validate the relations between the chemical ingredients and colour parameters for the tested Zedoray Rhizome samples, Pearson correlation analysis was performed. Generally, an absolute value of the correlation coefficient higher than 0.4 with a *p* value of less than 0.05 indicates a positive correlation between two variables. As shown in Table 5, for different species of Zedoray Rhizome samples, curzerene, furanodienon, demethoxycurcumin, and curcumin were positively correlated with parameters *L* and *b* but anticorrelated with the parameter *a*, whereas germacrone and isourecumenol were inversely related to parameters *L* and *b* but showed a positive correlation with the parameter *a*. Besides, curcumol was positively correlated with the parameter *L*. As for differently prepared slices of Zedoray Rhizome samples, curcumol was anticorrelated with the parameter *a*, while curdione, bisdemethoxycurcumin, and demethoxycurcumin were positively correlated with the parameter of *a*.

## 4. Conclusion

In this study, an intergrated approach that combined HPLC, colorimeter techniques, and chemometric tools was developed to evaluate the quality of Zedoray Rhizome samples from different species and differently prepared slices. The HPLC technique was applied to determine the contents of twelve chemical ingredients in different forms of Zedoray Rhizome samples, and the colorimeter was used to measure the colour parameters of *L*, *a*, and *b*. The chemometric tools, including PCA and Pearson correlation analysis, were employed to reveal the significant differences among the samples in terms of the contents of chemical ingredients and colour parameters and to explore the correlation between the chemical ingredients and the colour parameters. This approach provided a new path to the comprehensive quality assessment of Chinese herbs from the viewpoint of both the intrinsic (chemical) and extrinsic (appearance) level [20].

## Figures and Tables

**Table 1:** Optimization of extraction conditions.

Analytes	Extraction methods (%)		Extraction solvents (%)				Extraction time (%)		
	Sonication	Reflux	Methanol	80% aqueous methanol	60% aqueous methanol	Ethanol	30 min	45 min	60 min
Curcumenol	0.87	0.36	0.87	0.87	0.46	0.60	0.82	0.87	0.71
Curzerene	0.21	0.07	0.20	0.19	0.10	0.11	0.20	0.22	0.19
Curdione	1.96	1.22	1.96	0.61	0.58	1.66	1.86	1.96	0.88
Isourecumenol	0.73	0.40	0.69	0.38	0.32	0.68	0.74	0.74	0.38
Furanodienon	0.50	0.23	0.51	0.42	0.17	0.16	0.48	0.50	0.43
Curcumol	1.14	1.03	1.14	1.14	1.00	1.03	0.96	1.13	1.04
Germacrone	1.46	1.42	1.46	1.45	1.13	1.42	1.33	1.46	1.42
Furanodiene	0.25	0.20	0.25	0.23	0.17	0.21	0.14	0.25	0.21
*β*-Elemene	0.21	0.17	0.21	0.19	0.16	0.18	0.13	0.21	0.18
Bisdemethoxycurcumin	0.11	0.10	0.11	0.10	0.04	0.10	0.06	0.11	0.10
Demethoxycurcumin	0.37	0.35	0.37	0.37	0.18	0.36	0.30	0.36	0.35
Curcumin	0.20	0.19	0.20	0.19	0.09	0.19	0.15	0.20	0.19

**Table 2:** The results of methodology validation for HPLC analysis.

Analytes	Calibration curves	Range (*μ*g·mL^−1^)	LOD (*μ*g·mL^−1^)	LOQ (*μ*g·mL^−1^)	Recovery (%)	Precision (%)			Stability (%)
					Mean	RSD	Intraday	Interday	RSD
Curcumenol	*y* = 1.0 × 10^7^*x* − 56762 (*R*^2^ = 0.9993)	0.0037∼0.75	0.18	0.60	101.1	3.5	1.9	0.52	1.6
Curzerene	*y* = 2.0 × 10^7^*x* − 15711 (*R*^2^ = 0.9992)	0.0075∼1.88	0.06	0.21	99.24	2.4	2.5	1.3	2.0
Curdione	*y* = 2.0 × 10^7^*x* − 50917 (*R*^2^ = 0.9998)	0.0072∼2.71	0.15	0.50	100.4	1.8	0.28	1.6	1.8
Isourecumenol	*y* = 2.0 × 10^7^*x* − 11663 (*R*^2^ = 0.9997)	0.00032∼0.32	0.07	0.22	100.3	4.4	0.24	0.99	1.1
Furanodienon	*y* = 4.0 × 10^7^*x* − 6308 (*R*^2^ = 0.9998)	0.0069∼2.60	0.03	0.11	100.5	3.1	0.25	1.2	1.6
Curcumol	*y* = 2.0 × 10^7^*x* − 193973 (*R*^2^ = 0.9999)	40.00∼3590	0.17	0.57	99.98	2.1	0.23	0.97	1.2
Germacrone	*y* = 2.0 × 10^7^*x* + 812374 (*R*^2^ = 0.9998)	205.00∼1435	0.05	0.16	99.42	2.0	0.78	1.3	1.4
Furanodiene	*y* = 4.0 × 10^7^*x* − 19611 (*R*^2^ = 0.9999)	3.26∼1630.5	0.05	0.18	98.77	1.5	0.31	0.82	1.6
*β*-Elemene	*y* = 8.0 × 10^6^*x* − 21191 (*R*^2^ = 0.9999)	1.72∼724.5	0.33	1.08	99.95	1.9	1.8	1.4	2.1
Bisdemethoxycurcumin	*y* = 6.3 × 10^5^*x* − 4131.6 (*R*^2^ = 0.9998)	0.35∼4.16	0.06	0.11	98.57	2.0	0.45	1.4	0.49
Demethoxycurcumin	*y* = 9.8 × 10^5^*x* − 2.2 × 10^5^ (*R*^2^ = 0.9999)	0.72∼14.92	0.12	0.15	98.94	1.3	0.19	1.5	0.37
Curcumin	*y* = 1.3 × 10^6^*x* − 22070 (*R*^2^ = 0.9998)	0.26∼31.68	0.01	0.02	99.78	1.8	0.19	1.1	0.42

**Table 3:** Diagnostics of the PCA model for different species of Zedoray Rhizome samples.

Number of PCs	Eigenvalue	*R*_Xcum_^2^ (%)
1	7.55	50.3
2	3.89	76.2
3	1.84	88.5

**Table 4:** Diagnostics of the PCA model for differently prepared slices of Zedoray Rhizome samples.

Number of PCs	Eigenvalue	*R*_Xcum_^2^ (%)
1	5.14	34.3
2	2.81	53.0
3	1.77	64.8

**Table 5:** The Pearson correlation analysis of the colour parameters and the chemical ingredients.

Chemical ingredients	Different species			Differently prepared slices		
	*L*	*a*	*b*	*L*	*a*	*b*
Curcumol	0.480^*∗*^	0.389^*∗*^	−0.376^*∗*^	0.185	−0.420^*∗*^	0.383^*∗*^
Germacrone	−0.551^*∗*^	0.427^*∗*^	−0.455^*∗*^	−0.082	0.373^*∗*^	−0.300^*∗*^
Furanodiene	−0.359	0.242	−0.285^*∗*^	−0.025	−0.393^*∗*^	0.294^*∗*^
*β*-Elemene	−0.298^*∗*^	0.183	−0.213	−0.004	−0.354^*∗*^	0.379^*∗*^
Curcumenol	−0.258	0.171	−0.196	−0.173	−0.125	−0.022
Curzerene	0.646^*∗*^	−0.600^*∗*^	0.605^*∗*^	−0.171	−0.115	−0.245
Curdione	−0.389^*∗*^	0.296^*∗*^	−0.327^*∗*^	−0.155	0.448^*∗*^	−0.323^*∗*^
Isourecumenol	−0.408^*∗*^	0.432^*∗*^	−0.412^*∗*^	−0.108	0.084	−0.069
Furanodienon	0.609^*∗*^	−0.522^*∗*^	0.454^*∗*^	0.040	−0.270	0.042
Bisdemethoxycurcumin	0.272	−0.091	0.167	−0.254	0.402^*∗*^	−0.151
Demethoxycurcumin	0.540^*∗*^	−0.429^*∗*^	0.465^*∗*^	−0.091	0.520^*∗*^	−0.346^*∗*^
Curcumin	0.669^*∗*^	−0.617^*∗*^	0.625^*∗*^	0.315^*∗*^	−0.211	0.053

Notes: ^*∗*^refers to the *p* value less than 0.05.

## Data Availability

The data used to support the findings of this study are included in the article.

## Supplementary Materials

Supplementary Materials Table 1S: the details of collected Zedoray Rhizome samples. Table 2S: the details of CR and PCR samples.
