# Supplementary material for: Quality Assessment of Different Species and Differently Prepared Slices of Zedoray Rhizome by High-Performance Liquid Chromatography and Colorimeter with the Aid of Chemometrics
Source: J Anal Methods Chem. 2020 Sep 29;2020:8866250. doi: 10.1155/2020/8866250 (PMC7542477; doi:10.1155/2020/8866250)
Supplement: Supplementary Materials — Table 1S: the details of collected Zedoray Rhizome samples. Table 2S: the details of CR and PCR samples. [file 8866250.f1.docx]

## Supplementary Materials

Table 1S. The details of collected Zedoray Rhizome samples.

| Sample No. | Collection location | Collection date |
| --- | --- | --- |
| CW1 | Rui’an city, Zhejiang province | 2018.7.25 |
| CW2 | Rui’an city, Zhejiang province | 2018.7.25 |
| CW3 | Rui’an city, Zhejiang province | 2018.7.25 |
| CW4 | Rui’an city, Zhejiang province | 2018.7.25 |
| CW5 | Rui’an city, Zhejiang province | 2018.7.25 |
| CW6 | Rui’an city, Zhejiang province | 2018.7.25 |
| CW7 | Rui’an city, Zhejiang province | 2018.7.25 |
| CW8 | Rui’an city, Zhejiang province | 2018.7.25 |
| CW9 | Rui’an city, Zhejiang province | 2018.7.25 |
| CW10 | Rui’an city, Zhejiang province | 2018.7.25 |
| CW11 | Rui’an city, Zhejiang province | 2018.7.25 |
| CW12 | Rui’an city, Zhejiang province | 2018.7.25 |
| CP13 | Anyue city, Sichuan province | 2017.10.24 |
| CP14 | Anyue city, Sichuan province | 2017.10.26 |
| CP15 | Muchuan city, Fujian province | 2017.10.30 |
| CP16 | Chongzhou city, Sichuan province | 2017.10.30 |
| CP17 | Chongzhou city, Sichuan province | 2017.10.30 |
| CP18 | Sichuan province | 2017.10.30 |
| CP19 | Chongzhou city, Sichuan province | 2017.10.24 |
| CP20 | Sichuan province | 2017.10.26 |
| CP21 | Quanzhou city, Fujian province | 2017.10.30 |
| CP22 | Muchuan city, Fujian province | 2017.10.30 |
| CP23 | Quanzhou city, Fujian province | 2017.10.30 |
| CK24 | Yunfu city, Guangdong province | 2018.1.30 |
| CK25 | Guangxi province | 2018.1.26 |
| CK26 | Yulin city, Guangxi province | 2018.2.1 |
| CK27 | Yulin city, Guangxi province | 2018.4.2 |
| CK28 | Yulin city, Guangxi province | 2018.4.9 |
| CK29 | Yulin city, Guangxi province | 2018.4.9 |
| CK30 | Yulin city, Guangxi province | 2018.4.9 |
| CK31 | Yulin city, Guangxi province | 2018.4.9 |
| CK32 | Nanning city, Guangxi province | 2018.4.9 |
| CK33 | Myanmar | 2018.4.9 |
| CK34 | Myanmar | 2018.4.9 |
| CK35 | Myanmar | 2018.4.9 |
| CK36 | Vietnam | 2018.4.9 |
| CK37 | Vietnam | 2018.4.9 |
| CK38 | Vietnam | 2018.4.9 |
| CK39 | Qinzhou city, Guangxi province | 2018.5.2 |
| CK40 | Yulin city, Guangxi province | 2018.5.3 |
| CK41 | Beihai city, Guangxi province | 2018.5.4 |
| CK42 | Beiliu city, Guangxi province | 2018.5.7 |
| CK43 | Yulin city, Guangxi province | 2018.5.10 |
| CK44 | Chongzuo city, Guangxi province | 2018.5.10 |
| CK45 | Yulin city, Guangxi province | 2018.5.10 |
| CK46 | Yulin city, Guangxi province | 2018.5.10 |
| CK47 | Yulin city, Guangxi province | 2018.5.10 |
| CK48 | Yulin city, Guangxi province | 2018.5.10 |
| CK49 | Yulin city, Guangxi province | 2018.5.10 |
| CK50 | Nanning city, Guangxi province | 2018.5.10 |

Table 2S. The details of CR and PCR samples.

| CR | PCR | Origin | Collection location | Collection date |
| --- | --- | --- | --- | --- |
| CR1 | PCR1 | Guangxi | Bencaotang TCM slice factory | 2018.4.25 |
| CR2 | PCR2 | Guangxi | Bencaotang TCM slice factory | 2018.4.25 |
| CR3 | PCR3 | Guangxi | Kangmei TCM slice factory | 2018.5.10 |
| CR4 | PCR4 | Guangxi | Kangmei TCM slice factory | 2018.5.10 |
| CR5 | PCR5 | Guangxi | Kangmei TCM slice factory | 2018.5.10 |
| CR6 | PCR6 | Guangxi | Guokang TCM slice factory | 2018.3.13 |
| CR7 | PCR7 | Guangxi | Hexiang pharmaceutical co. LTD | 2018.4.16 |
| CR8 | PCR8 | Guangxi | Bencaotang TCM slice factory | 2018.7.25 |
| CR9 | PCR9 | Guangxi | Bencaotang TCM slice factory | 2018.7.25 |
| CR10 | PCR10 | Guangdong | Tiancheng TCM slice factory | 2018.8.8 |
| CR11 | PCR11 | Sichuan | Yudingtang TCM slice factory | 2018.7.25 |
| CR12 | PCR12 | Guangxi | Zhongqiang TCM slice factory | 2018.7.10 |
| CR13 | PCR13 | Guangxi | Jinye pharmaceutical company | 2017.10.24 |
| CR14 | PCR14 | Fujian | Xinyuan TCM slice factory | 2017.12.26 |
| CR15 | PCR15 | Guangxi | Yulin harmony pharmaceutical | 2017.10.30 |
| CR16 | PCR16 | Guangxi | Tongrentang Chinese Medicine since 1669 | 2017.11.3 |
| CR17 | PCR17 | Guangxi | Guangxi harmony pharmaceutical company | 2017.11.7 |
| CR18 | PCR18 | Fujian | Xinyuan TCM slice factory | 2017.10.30 |
| CR19 | PCR19 | Guangxi | Raybo TCM slice factory | 2017.10.24 |
| CR20 | PCR20 | Guangdong | Tiancheng TCM slice factory | 2017.10.26 |
| CR21 | PCR21 | Guangdong | Yudingtang TCM slice factory | 2017.10.30 |
